# Report of the Medical Department of the Board of Education

**Published:** 1917-10-27

**Authors:** 


### REPORT OF THE MEDICAL DEPARTMENT OF THE BOARD OF EDUCATION.

Sir George Newman has a large field to survey, and what he has to tell us is of the highest national interest. In this acute hour he can hardly fail of an attentive audience, for patriotism not less than human sympathy is engaged in the story. Whatever of strength or virtue is possible for the future of our country lies in the efficient care and training of the school-children of to-day. It is here that the hope and promise of the nation are to be found. That these children should be brought under the influence of systematic medical inspection and guidance is a comparatively new notion. The School Medical Service only dates from 1907, and in its early days was by no means free from opposition. Since its origin it has been developed and extended, and not much is heard of the critics nowadays. From the discovery of ailments and defects and disabilities in the school-children other questions have necessarily emerged. The most immediate and obvious one is that of the treatment of the sufferers, and this touches nice points in relation to parental authority and responsibility and difficulties associated with medical organisation. Another problem is that of the prevention of the ills which handicap the child as a scholar, and this invades the period prior to the school age; it comes close to the problems of maternity and the nurture of the infant.

### Important Outstanding Questions.

Physical training as well as scholastic achievement is also in issue, and for obvious reasons is receiving an increasing recognition. Nor can the question of adolescence be excluded, for much of what may be gained at school will inevitably be lost—alike on the mental, the moral, and the physical side—unless means are taken to secure it. Such are the larger and more outstanding questions which face the Medical Department of the Board of Education, and Sir George Newman does not avoid any of them. Some of them are already within the compass of the law, and can be met by organisation and administration given the necessary central or local driving force. Others must look to a ripening public opinion to bring them within the legislative scheme. But all are discussed in the Report with a fullness of knowledge and with a frankness which gives to the document a highly engaging quality. The private citizen, not less than those with larger responsibilities, may be urged to study the Report as a serious contribution to a subject of the highest practical and patriotic interest.

### The Child's Natural Right to Health.

The verdict which Sir George Newman pronounces after comparing the present position with that of earlier years is—"There are, happily, signs of substantial progress." The School Medical Service has won a large measure of recognition, and hundreds of thousands of children are healthier, brighter, and better for its labours. Objections have diminished almost to the vanishing point, and the natural right of the child to health and well-being is admitted in an ever-increasing degree. Even in time of war public opinion on this point has shown itself sufficiently strong and stable to enforce the maintenance of at least a minimum of medical services, and this experience may be taken as a rough measure of the popular appreciation which these services now command. The war has limited the work and has interfered with its development. But some claims for its complete suspension failed to command support. Evidently the conviction is firmly established in the public mind that in

REPORT OF THE MEDICAL DEPARTMENT OF THE BOARD OF EDUCATION—(*continued*).

the process of school education, and in the problems which grow out of this, medical supervision and direction must play a large and increasing part.

One Million Children Need Treatment.

That there is ground for this conviction the facts amply demonstrate. Stated broadly, the position is that out of some 7,000,000 children of school age, not less than a million are physically or mentally defective or diseased to such a degree as to be unable to derive reasonable benefit from the education which the State provides. A large proportion of these evils may certainly be relieved or removed by appropriate medical treatment, and it is therefore little wonder that among the conditions which Sir George Newman prescribes as the irreducible minimum of his demands is to be found the claim "that for every sick, diseased, or defective child skilled medical treatment shall be made available, either by the local education authority or otherwise." At present the local authorities are in this respect not under legislative pressure, and some of them, for one reason or another, leave the question of medical treatment for the children under their charge severely alone. That such a position will be allowed to continue it is impossible to believe. Nor can it be denied that the question is one in which the responsibility of the medical profession is directly engaged. It is the duty of the profession to help to educate public opinion on the question. Equally it is a fair demand that the profession shall be ready to undertake the work on fair and reasonable conditions. The official conditions have not always possessed these qualities, but there are, we gladly recognise, evidences of improvement. The work is done in the national interest, and it ought to be recognised and treated on this basis. The "school clinic" is establishing itself as a necessary part of the educational machinery, and the nation, it is certain, will not permit any halt to be called. The mere enumeration of ailments and defects as a cold-blooded statistical record cannot be treated with patience unless attached to it is an organisation directed to the suppression of these evils.

Sir George Newman's Ideal.

Immediately, this organisation must deal with individual sufferers, but the time must come when the earlier and more effective measures of prevention must take a prominent place; and similarly, the child, protected, educated, and developed at the expense of the State, cannot, on mere grounds of economy, be allowed to fail in the possibility of good citizenship for want of mental and physical opportunity in the years which immediately follow his escape from continuous school attendance. How far compulsion is good for the adult is a highly debateable question, but no one doubts its necessity for the child if he is to prove himself "a human personality, well grown and ready in body and mind, able to work, able to play, a good citizen, the healthy parent of a future generation." This is Sir George Newman's ideal, and we shall make no attempt to better it.

